# Quality of diabetes care at Armed Forces Hospital, Southern Region, Kingdom of Saudi Arabia, 2006

**DOI:** 10.4103/1319-1683.74328

**Published:** 2010

**Authors:** Ibrahim S. Al-Arfaj

**Affiliations:** *Diabetes Center, Armed Forces Hospital, Khamis Mushate, Kingdom of Saudi Arabia*

**Keywords:** Armed forces hospital, care, diabetes, quality, southern region

## Abstract

**Objective:**

The aim of this study was to assess the current status of care provided by the Diabetes Center at Armed Forces Hospital, Southern Region.

**Materials and Methods:**

A total of 260 patients were randomly selected from the diabetic patients attending the Diabetes Center. Study tools comprised patients’ data sheets and patients’ interview questionnaire.

**Results:**

Two-thirds of the patients were aged 50 years or more. Half of patients had had the disease for less than 10 years. Diet therapy alone was followed by 2.3% of diabetic patients. More than half of patients (56.5%) were on insulin. Most of the diabetic patients were tested for HbA1c at least once per year (88.1%), and 71.5% had their lipid profile done at least once within two years. Low indicators included having a dilated eye examination (35.4%), assessment for nephropathy (28.8%), and having a well-documented foot examination (12.7%). Highest risk HbA1c level (>9.5%) was reached by 38.8% of patients, 48.8% had a low-density lipoprotein level of <130 mg/dl, and 36.5% of patients had controlled blood pressure (≤130/80 mmHg). Most patients were satisfied with their interaction with the treating doctor, 41.5% were satisfied with access to treatment. Hypertension was found to be the most frequent comorbidity (38.5%).

**Conclusion:**

The quality of services as regard to process and outcome are low at the Diabetes Center. The overall diabetic patients’ satisfaction was high, whereas their satisfaction was low as regards to access to treatment or health professionals.

## INTRODUCTION

Results obtained from clinical trials over the past decade have led to the provision of guidelines that advocate aggressive management of hyperglycemia, hypertension, and hyperlipidemia for patients with diabetes.[1–7] Further research has established the evidence base for specific screening and prophylactic recommendations, including retinal and foot examination and daily aspirin.[8–10] Despite the scientific progress, patients with diabetes continue to suffer from high rates of cardiovascular and microvascular complications and can expect a reduction of their lifespan by 10 to 15 years.[11,12]

In 2001, the Diabetes Quality Improvement Project (DQIP) was initiated in USA to define a comprehensive set of measures for evaluating the quality of diabetes care.[13] The DQIP measures are indicators or tools to assess the level of care provided within systems of care to populations of patients with diabetes.[14]

A Diabetes Center was established in June 2004 at the Armed Forces Hospital, Southern Region (AFHSR), KSA, to improve the diabetes services by providing a comprehensive, continuous, and evidence-based medical care. Since then, there has been no internal or external assessment of the quality of service provided by the center. This study intends to provide information that will help to improve quality internally, and provide measurements for comparison with other diabetes health care services elsewhere in the Kingdom or internationally to improve accountability. The aim of this study is to assess the current status of care provided by the AFHSR Diabetes Center, KSA.

## MATERIALS AND METHODS

This cross-sectional study was conducted at the Armed Forces Hospital in Khamis Mushate, Southern Province, in June 2006. In the Diabetes Center, clinics provide both primary and specialty care for diabetic patients. Approximately 3500 diabetic patients visit this center. Two internal medicine specialists cover the primary health care clinics, and two endocrinologists cover the specialty clinics. There are also two nurses, two female dietitians, one female health educator, and one podiatrician.

During the month of June, there were 673 prearranged bookings with primary and specialty clinics. A total of 260 diabetic patients who had been seen in the center in three or more visits were randomly selected from the total booking list using the simple random technique. Patients’ records were reviewed by using the checklists (appendix A). Selected patients were interviewed by the researcher using a questionnaire designed by the DQIP. This included patient’s identification data in addition to self-management, health and nutrition education, interpersonal care from provider, satisfaction with, and access to care, health status, and counseling on cessation of smoking. A five-grade scaling system (very satisfied, moderate satisfaction, satisfied, poor satisfaction, dissatisfied) was developed by the researcher and used to assess the previous satisfaction indicators. The researcher filled the questionnaire during interview with the diabetic patients attending the Diabetes Center, or the accompanying relatives for dependent patients.

According to the DQIP initial measure set,[14] the process and outcome indicators were used to evaluate the process and outcome of services provided by the Diabetes Center (Appendix A). Additional indicators to the outcome evaluation are the prevalence rates of complications (i.e., myocardial infarction, nephropathy, retinopathy, neuropathy, and peripheral vascular diseases).

All the necessary official permissions were fully obtained before data collection. Collected data were verified before computerized data analysis. The Statistical Package for Social Sciences (SPSS ver. 13.0) was used for that purpose. Descriptive statistics (e.g., frequency and percentage) and Chi-square to test correlation between independent variables were calculated.

## RESULTS

The total number of diabetic patients who participated in this study was 260 and Table 1 shows their characteristics. HbA1c was tested once per year at least in 88.1% of diabetic patients and only 8.1% of them attained HbA1c level less than 7%. Only 36.5% of patients attained the targeted BP (<130/80 mmHg). Table 2 shows the initial measure set, process, and outcome indicators among diabetic patients according to DQIP. Female patients had HbA1c testing, LDL-C testing, dilated eye exam, and detailed foot exam more than male patients (*P* = 0.05). In male diabetic patients, the highest risk HbA1c was significantly more frequent (*P* = 0.005); there was no significant difference between male and female diabetics in the control of LDL-C and blood pressure.

**Table 1:** Patients’ socio-demographic and clinical characteristics, Diabetes Center, Armed Forces Hospital, Southern Region, Kingdom of Saudi Arabia, 2006

Characteristics (n = 260)	No. (%)
Sex	
Males	125 (48.1)
Females	135 (51.9)
Age group (in years)	
<30	34 (13.1)
30−49	59 (22.7)
50−69	131 (50.4)
70+	36 (13.8)
Patients’ cigarette smoking status	
Nonsmoker	257 (98.8)
Smoker	3 (1.2)
Body mass index	
Normal (<25 kg/m^2^)	57 (22)
Overweight (25−29.9 kg/m^2^)	73 (22)
Obese/morbid obese (>30 kg/m^2^)	130 (50)
Type of diabetes	
Type 1	34 (13.1)
Type 2	226 (86.9)
Duration of diabetes (in years)	
<10	127 (48.8)
10+	133 (51.1)
Type of therapy	
Diet	6 (2.3)
Oral hypoglycemics	166 (63.8)
Insulin	147 (56.5)

**Table 2:** Diabetes Quality Improvement Project initial measure set, process and outcome indicators among diabetic patients at Diabetes Center, Armed Forces Hospital Southern Region, Kingdom of Saudi Arabia, 2006

Process Indicators (n = 260)	No. (%)
Patients receiving >1 HbA1c test/year	229 (88.1)
Patients assessed for nephropathy	75 (28.8)
Patients receiving a lipid profile once in 2 years	186 (71.5)
Patients receiving a dilated eye examination	92 (35.4)
Patients receiving a well-documented foot	33 (12.7)
examination	
Outcome indicators (n = 260) HbA1c level distribution	
Not tested	20 (7.7)
<7	21 (8.1)
7−7.9	42 (16.2)
8−8.9	48 (18.5)
9−9.9	39 (15)
>9.9	90 (34.6)
Systolic blood pressure distribution (mmHg)	
<140	159 (61.2)
140−159	72 (27.7)
160−179	25 (9.6)
180−209	4 (1.5)
Diastolic blood pressure distribution (mmHg)	
<90	253 (97.3)
90−99	5 (1.9)
100−109	2 (0.8)
LDL-C level distribution	
<100	72 (27.7)
100−129	56 (21.5)
130−159	44 (16.9)
>159	18 (6.9)
Degree of control	
Highest risk HbA1c level (>9.5%)	101 (38.8)
Patients with a low-density lipoprotein <130 mg/dl	127 (48.8)
Patients with blood pressure <140/90 mmHg	158 (60.8)
Patients with blood pressure <130/80 mmHg	95 (36.5)

Of the patients, 83.5% received health education at the Diabetes Clinic. However, 79.6% had understood the instructions given and only 55% had followed them. Two-third of diabetic patients (63.5%) were involved in their health care decisions, 91.2% of them were satisfied with their interaction with the treating doctor, 41.5% were satisfied with access to treatment or health professionals, while 89.2% were generally satisfied.

**Figure 1:**
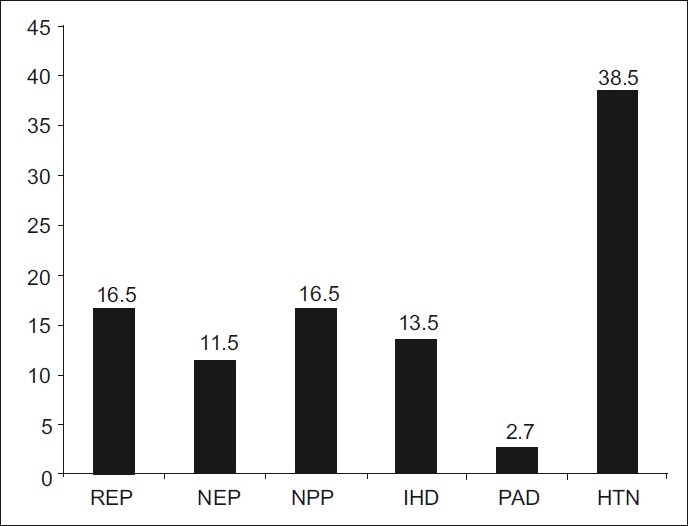
Frequency of diabetes complications and comorbidities among diabetic patients at Diabetes Center, Armed Forces Hospital, Southern Region, Kingdom of Saudi Arabia, 2006

Figure 1 shows comorbidities or complications among diabetic patients. Hypertension was the most frequent (38.5%) complication.

## DISCUSSION

Improved blood glucose control, regular eye examinations, and reduction in cholesterol and blood pressure are some of the practices that have been unequivocally shown to reduce complications, and thereby diminish the heavy personal and financial toll of diabetes.[15] The aim of the present study was to describe the current status of care provided by the AFHSR Diabetes Center.

The study indicated that there were slightly more female diabetic patients than males, two-third of whom were aged 50 years or more and half of whom had had their disease for less than 10 years. This was in agreement with several national and international studies.[16–19] El-Hazmi *et al*. noted that the increase in prevalence of diabetes in those aged 45 years and above was very significant in the Saudi population, and placed Saudi Arabia among those countries of world classified as high-prevalence countries. Differences in gender-specific prevalence rates is possibly due to the differences in the lifestyles of the men and women population.[19]

Results of the present study showed that less than one-fourth of the patients (22%) had normal weight. Some studies have emphasized the significance of the high prevalence of obesity in the Saudi population as a risk factor for diabetes. El-Hazmi *et al*., Al-Owayyed *et al*., and Al-Alfi *et al*. noted that in KSA, overweight and obesity are common in both men and women.[18–21] These findings are even higher than what has been reported in several studies, and by Grant *et al*. who conducted a retrospective study in USA. They noted that obesity is highly prevalent among American diabetic patients (31.2%).[17]

Valk *et al*. noted that the main risk factors contributing to the increasing incidence of type 2 diabetes are the unrelenting rise in obesity and physical inactivity.[22]

The low rate of patient on diet therapy alone was comparable with those reported by Grant *et al*. who stated that of those patients attending the Diabetes Clinics, 2.7% were on diet therapy only, 30.2% were on hypoglycemic therapy, while 67.1% received insulin therapy.[17] In Amsterdam, the study of Valk *et al*. revealed that the percentage of patients who were on diet only management decreased from 31.2% in 1992 to 8.3% in 1996.[22] This could be explained by the current recommendations on the importance of aggressive control of blood sugar, lack of patient adherence to diet and exercise advice, or the absence of clear practice guideline provided at the center to emphasize the role of nonpharmacological interventions.

The present study revealed that the DQIP process indicators were quite low. Al-Owayyed *et al*. in Riyadh got better results of process indicators for lipid profile testing, dilated eye examination, and foot examination. They were 73.8, 61.5, and 53.3%, respectively. Other process indicators were 60.5% for HbA1c testing and only 12% for microalbuminuria.[18]

Grant *et al*. revealed a higher process indicator for HbA1c which was measured for 98.8% of American diabetic patients, Lipid profile was measured for 86.9% of diabetic patients, dilated eye examination performed for 55.4% of patients, screening for nephropathy for 65.1% of patients, while there were documentation for foot examination for 63.6% of diabetic patients.[17]

Several factors might contribute to these findings. These include poor patient compliance to advice, treatment or appointments, heavy workload at clinics, absence of practice guidelines, or lack of self care and effective health education programs. However, the actual causes must be explored and managed accordingly.

Furthermore, the outcome indicators were also low. The present study showed that the highest risk HbA1c level (>9.5%) was reached by 38.8% of diabetic patients, while only 8.1% of diabetic patients attained HbA1c level less than 7%. In USA, Grant *et al*. showed that this outcome indicator (HbA1c level less than 7%) was much better attained by one-third of diabetic patients (34%).[17]

Previous low indicators found in this study can be explained by several factors like the lack of adherence to practice guidelines by the practitioners, patients’ noncompliance to advice, treatment, or appointments, or simply missing records for patients’ workup.

Moreover, the present study showed that 48.8% of diabetic patients had low-density lipoprotein (<130 mg/dl). A comparable level for this indicator was reported by Grant *et al*. on American diabetics (52.9%).[17]

Blood pressure control (≤130/80 mmHg) among diabetic patients was attained by 36.5% of diabetic patients. This result is lower than that achieved in the American study by Grant *et al*., which reported controlled blood pressure in 55% of diabetic patients.[17]

This study showed that 83.5% of diabetic patients were given health education. However, only 79.6% were able to understand the instructions and only 55% of the diabetic patients followed them. This discrepancy can be explained by the fact that the health educator for diabetic patients in the Diabetes Clinic at the AFHSR was a woman, as has been explained by several authors. Elasy *et al*. emphasized that diabetes education was an essential part of diabetes care. However, problems with communication and cultural differences may hinder delivery of the best diabetes care to different ethnic groups.[23] In Turkey, Uitewaal *et al*. noted that the influence of gender bias in favor of men might explain why the advice and suggestions on life style changes given by female educators had little effect on the male patients. Male patients felt less inclined to take advice on behavioral changes from women.[24]

Austin[25] stated that diabetes education is usually underutilized. Approximately 60 to 70% of patients with diabetes have no instruction on self-management. Diabetes educators should be trained to identify and help overcome barriers in order to provide the best care. He advised that educators must base their intervention on the following seven self-care behaviors: (1) healthy eating, (2) being active, (3) monitoring, (4) taking medication, (5) problem-solving, (6) healthful coping, and (7) reducing risks.

The present study showed that two-thirds of diabetic patients (63.5%) were involved in the decisions on their health care. Anderson *et al*. stated that substantial proportion of diabetic patients report difficulty in reaching the goals set for self-care treatment. They described the unmet need for the knowledge and skills of diabetes self-care associated with patient outcomes. Routine monitoring of patient-centered self-care outcomes could help improve long-term outcomes of diabetic care.[26]

The percentage of patients who were generally satisfied was 89.2%, and 91.2% of the diabetic patients were satisfied with their interaction with the treating doctor. However, only 41.5% were satisfied with access to treatment or health professionals. This finding can be explained by the crowded appointment schedule, shortage of staff, or lack of other means of communication. However, there should be a study to find out the real reasons behind the lack of satisfaction with access to treatment.

The study indicated a low (1/2%) prevalence of cigarette smoking among diabetics. This finding is in agreement with that noted by Harris *et al*. who stated that people with diabetes who smoke had a substantially increased risk of cardiovascular disease, above and beyond that attributed to diabetes itself. The cessation of smoking was the most important and effective way of reducing diabetes-related morbidity and mortality in smokers.[27] This finding was much lower than that reported by Al-Owayyed *et al*., which was 12.9%.[18]

The present study revealed that hypertension was the most frequent comorbidity among diabetic patients (38.5%). This was comparable with that reported by Al-Owayyed *et al*. in Riyadh, which found hypertension in 31% of the patients, retinopathy in 17.9%, nephropathy in 13.3%, ischemic heart disease in 6.6%, and neuropathy in 4.8%.[18]

In Canada, Hanley *et al*. noted that the high-prevalence rates of both micro- and macroalbuminuria among diabetics explained the high incidence of renal complications of diabetes. They reported a high prevalence of neuropathy in diabetics (46.3%), followed by retinopathy (24%).[28]

Differences in reported complications attributable to diabetes, as assessed by the DQIP process and outcome indicators may reflect differences both in duration or severity of disease, in addition to differences in quality of health care provided for diabetic patients.[27]

## CONCLUSIONS

The quality of service given to diabetic patients in the diabetes center and the outcomes were low, despite the high level of patient satisfaction with health care team. In order to improve the quality of diabetic care in the center, health education and self-care management should be positively promoted. However, the presence of a plan that describes the steps of overcoming the barriers & improving the compliance of both physicians & patients to national & international guidelines recommendations is necessary.

## Appendix A: The DQIP initial measure set, the process and outcome indicators[14]

**Process indicators**

1. Percentage of patients receiving >1 HbA1c test/year
2. Percentage of patients assessed for nephropathy
3. Percentage of patients receiving a lipid profile once in 2 years
4. Percentage of patients receiving a dilated eye exam
5. Proportion of patients receiving a well-documented foot exam to include a risk assessment

**Outcome indicators**

1. HbA1c levels of all patients reported in six categories (i.e.,<7.0%, 7.0-7.9% 8.0-8.9%, 9.0-9.9%, >10.0%, no value documented)
2. Distribution of blood pressure values (i.e., <140, 140-159, 160-179, 180-209, >209 mmHg systolic; <90, 90-99, 100-109, 110-119, >119 mm Hg, no value documented)
3. Distribution of LDL values (i.e., <100, 100-129, 130-159, >159 mg/dl, no value documented)
4. Percentage of patients with the highest risk HbA1c level (i.e., HbA1c >9.5%)
5. Percentage of patients with a low-density lipoprotein (LDL) <130 mg/dl
6. Percentage of patients with blood pressure <140/90 mmHg
